# Biomonitoring of a Nile Delta Lake using benthic foraminifera

**DOI:** 10.1007/s10661-022-10611-w

**Published:** 2022-11-07

**Authors:** Ahmed M. BadrElDin, Khairia M. Al-Qahtani, Nadia B. E. Badr

**Affiliations:** 1 Faculty of Science, Department of Oceanography, Alexandria University, Alexandria, 21511 Egypt; 2 Princess Nourah Bint Abdulrahman University, Riyadh, Saudi Arabia; 3 Faculty of Science, Department of Environmental Science, Alexandria University, Alexandria, 21511 Egypt

**Keywords:** *Ammonia tepida*, Bioindicator, Heavy metals, Pollution, Foraminiferal abnormality index, Egypt

## Abstract

Lake Edku, one of the northern Nile Delta lakes, is a shallow brackish coastal lake subjected to domestic and agricultural effluents via two main drains, El-Khairy and Barsik, in addition to the discharge water of hundreds of fish farms. This study measures the responses of the benthic foraminiferal assemblage to the environmental stressors in Lake Edku. Grain size, organic carbon, and seven potentially toxic elements (Cu, Pb, Zn, Cd, Cr, Ni, and As) were determined in nine short cores (25–35 cm depth). The lake was characterized by vertical increase in mud, organic matter, and concentrations of all metals, particularly in the eastern basin at the vicinity of the drain discharges. This trend coincides with a general decrease in species diversity and increase in deformed specimens. The foraminiferal assemblage was dominated by *Ammonia tepida* (Cushman), a pollution-tolerant and euryhaline species. This study demonstrates that benthic foraminiferal assemblages provide a reliable pollution proxy in the brackish environments of Nile Delta that can be used in the periodical monitoring of the coastal lakes.

**Supplementary Information:**

The online version contains supplementary material available at 10.1007/s10661-022-10611-w.

## Introduction

The Egyptian government, with its ambitious strategies for the future, plans to protect and conserve the coastal ecosystems for sustainable development. Goal #14 of the Egyptian National 2030 Agenda is “conserve and sustainably use of the oceans, seas, and marine resources.” Decision makers critically need fundamental data regarding the current conditions and predictions of future changes in ecosystems quality, to determine and mandate precautions regarding the hazardous effects of environmental deterioration. Lake Edku is a human-impacted Nile Delta Lake on the northern Mediterranean coast of Egypt. It supports a fishery that accounts for more than 5% of the Egyptian northern lakes fish production and provides habitat for both wintering and breeding water birds (Khalil et al., 2013). The environment of the lake has been substantially degraded after the construction of Aswan High Dam over the Nile River in 1965 (Zalat & Vildary, 2007) as it resulted in (1) checking the flow of water downstream, (2) drastic changes in the physico-chemical and biological parameters of the Nile ecosystem, and (3) the reduction in area of three critical lacustrine ecosystems of the Nile Delta including Edku Lake (Abdel-Satar et al., 2017; El-Shazly, 2019). The input of untreated wastes from many local pollution sources (agricultural, industrial, and urban effluents) along its eastern and southern margins was an additional threat on Edku Lake’s environmental quality (Badr & Hussein, 2010). Dickman (1998) concluded that assessments of the ecological quality of aquatic ecosystems should not depend on chemical measurements only, yet pollutant impacts can be observed directly by studying the affected biological communities.

Benthic foraminifera (BF) are single-celled microscopic organisms (Kingdom Protista), many of which construct shells (commonly called tests) made of calcium carbonate or by agglutinating sediment particles. Because BF are extremely diverse and cosmopolitan, some species can tolerate in almost any marine environment (Förderer et al., 2018). With relatively short life cycles, BF can respond quickly to changes in physical and geochemical factors including temperature, salinity, pH, oxygen variability, sediment texture, organic carbon, and inorganic sediment composition, through changing foraminiferal assemblages (Murray, 2006). In addition, changes in their community structure (e.g., assemblages) have been widely used as a tool to help in the interpretation and reconstruction of modern and ancient environments (e.g., de Jesus et al., 2020; Narayan et al., 2015; Reymond et al., 2013). An added advantage of BF is their high preservation potential in the sediment record and the abundance of their tests that provides comparative information to assess short-term environmental changes (years to decades). The responses of BF to environmental stress have been used for more than 60 years as indicators for characterization and monitoring anthropogenically impacted coastal systems (e.g., Ben-Eliahu et al., 2020; Chalkley et al., 2019; Dimiza et al., 2019; Resig, 1960). Benthic foraminifera respond to both natural and anthropogenic environmental gradients as evidenced in taxonomic structure, foraminiferal density and diversity, and increased occurrences of deformities (e.g., Coccioni et al., 2009; Elshanawany et al., 2011; Martins et al., 2016; Samir & El-Din, 2001; Yanko et al., 1998).

The main objectives of the present work were to (1) investigate the species composition and diversity of the benthic foraminiferal assemblages and (2) determine the foraminiferal assemblage response to wastewater discharges, including organic-carbon and heavy metals. Two ecological proxies, species richness and foraminiferal abnormality index (FAI), were utilized as environmental stress indicators.

## Materials and methods

### Data collection

**Fig. 1:**
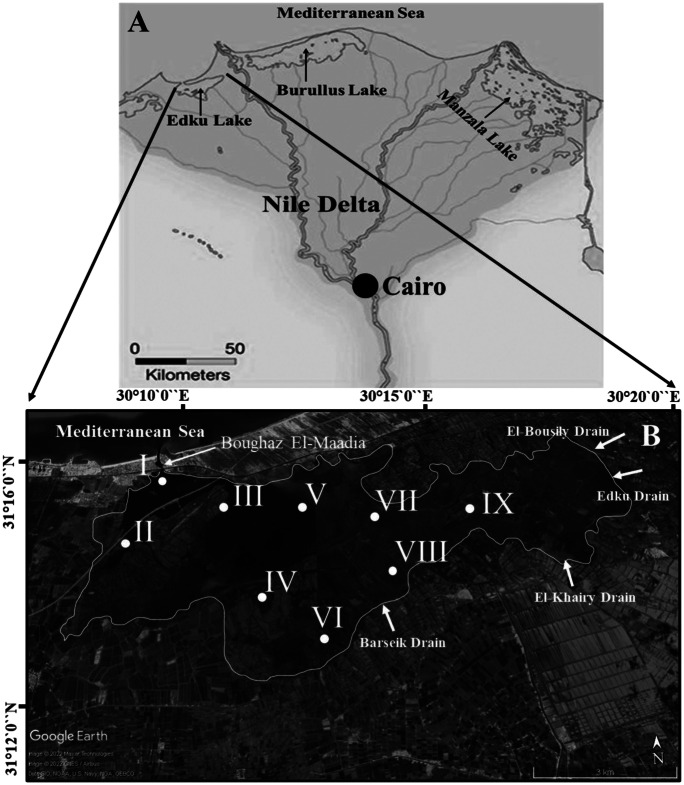
**A** Map showing location of Edku Lake northeast Nile Delta of Egypt. **B** Google map showing the sampling locations after BadrElDin et al. (2022)

Lake Edku is the third-largest, shallow-brackish coastal basin in northern Egypt (Fig. 1), situated west of the Nile Delta (latitudes 31° 11′ 30″ and 31° 18′ 00″ N, longitudes 31° 8′ 30″ and 31° 23′ 00″ E) and separated from the Mediterranean Sea a coastal by sand barrier. A narrow, 2 m depth entrance (Boughaz El-Maadia) on the west side allows hydrodynamic circulation between Lake Edku and Abu-Qir Bay. Abdel Halim et al. (2013) reported a very low average salinity (1.13%o ± 0.19) in the eastern basin of the lake, which is affected by discharges from the El-Khairy drain. Seawater enters the lake from Abu-Qir Bay through Boughaz El-Maadia, increasing the salinity to about 15‰ in the lake entrance (Abdel Halim et al., 2013). The water in the lake varies from clear to very turbid with sediment and plankton where its water depth ranges from 40 to 150 cm with an average of ~ 1 m (Abdallah, 2017). Lake Edku is divided into three main basins: western, central, and eastern. Two main drains; El-Khairy drain, which connects to three subdrains (Edku, El-Bousily and Damanhour), and the Barsik drain (Fig. 1), discharge domestic, agricultural, and industrial wastewater (Ossman & Badr, 2010), as well as the drainage water of more than 300 fish farms, into middle and eastern lake basins (Badr & Hussein, 2010). The lake receives total annual untreated drainage water (domestic, agricultural, and industrial) of 592 × 10^6^ m^3^/year and 348 × 10^6^ m^3^ from El-Khairy and Barsik drains, respectively (Morsy et al., 2020).

Short sediment cores were collected in triplicate in 2020 from nine sites; one replicate was used for sedimentological and geochemical analyses and two replicates for foraminiferal assessment (Fig. 1). The sampling was undertaken using heavy duty PVC tubes (5 cm diameter and 50 cm height). The sediment cores were carefully extruded, preserved in clean labeled polyethylene bags, and were kept in ice tank until arrival at the laboratory. As the core samples lack any kind of stratification, they were sliced at 5-cm intervals to yield three or four subsamples (0–5 cm, 5–15 cm, 15–25 cm, and 25–35 cm).

### Geochemical and contamination evaluation data

The mechanical sieving and pipette analyses were applied for grain size determination (Folk, 1980), and the percentages of sand (S%), silt (Z%), and clay (C%) were calculated. The total organic carbon (TOC%) was determined by loss in ignition (Heiri et al., 2001), and the total carbonate (TCO_3_%) was determined by the indirect method (Vogel, 1978). Concentrations of seven elements were analyzed (Cu, Pb, Zn, Cd, Cr, Ni, and As) using the method described by Liao et al. (2014). Three environmental quality indices were employed to evaluate sediment contamination: (1) contamination factor (CF) (Pekey et al., 2004), (2) degree of contamination (DC) (Håkanson, 1980), and (3) sediment quality guidelines (SQG) (Long et al., 1995; MacDonald et al., 2000). The methodologies used to determine the environmental variables and to calculate the environmental quality indices were fully described in BadrElDin et al. (2022). The base values that correspond to the terminologies used to describe the CF and DC are presented in Table 1.

**Table 1:** Terminology of pollution classes for contamination factor (CF) and degree of contamination (DC)

Contamination factor^a^		Degree of contamination^b^	
CF	Pollution	DC	Pollution
< 1	Low	< 8	Low
1 –3	Moderate	8–16	Moderate
3–6	Considerable	16–32	Considerable
≥ 6	Very high	≥ 32	Very High

^a^Contamination factor (CF) classes after Qingjie et al. (2008)

^b^Degree of Contamination (DC) classes after Håkanson (1980)

### Foraminiferal data

For all samples, constant volumes of 50 cm^3^ were washed over 63-μm sieves, and coarse fractions were oven-dried at 50 °C. Samples were examined using a stereomicroscope, and ~ 100 foraminiferal tests were handpicked from each sample (the foraminiferal fauna of Lake Edku was made up of very few species; see also Fatela and Taborda (2002)). Specimens were identified to genera level following the generic classification of Loeblich and Tappan (1988) and to species level consistent mainly with Cimerman and Langer (1991) and a variety of sources. The name of each species was checked and revised in accordance with the online database WoRMS (World Register of Marine Species; Hayward et al., 2020). Species richness and foraminiferal abnormality index (FAI, Coccioni et al., 2005) were evaluated. Selected species and deformed specimens were photographed using scanning electron microscope (SEM) JSM–IT 200 in Faculty of Science, Alexandria University.

### Statistical analyses

Species counts in each sample were converted to percent abundance (i.e., species relative abundance). The data set was the averages of the species relative abundance, and all determined environmental variables for each individual core and its related salinity (Table 2). The salinity data was provided by Prof. Nadia B.E. Badr (personal communication). The results of CF, DC, and FAI were not included in the statistical analyses. Analysis was applied to taxa that averaging ≥ 5% relative abundance in at least one core in the subsequent statistical analyses (Fishbein & Patterson, 1993). The infrequently occurring taxa do not significantly affect the formation of the major groups (Frezza & Carboni, 2009; Romano et al., 2008), and focusing on the most abundant taxa reduces background noise and reveals the underlying signatures of the assemblages (Fajemila et al., 2020). The data set was normalized using the equation (*N* = (value-mean)/standard deviation). One-way ANOVA analysis was performed to show the variability between the cores using Past program (V. 4.03). Q-mode cluster analysis (QCA) and principal component analysis (PCA) were carried out to identify cores characterized by similar assemblages and illustrate the key factors controlling the distribution of BF in core sediments (Chai et al., 2017; Jiang et al., 2019). The QCA and PCA analyses were performed using the program of SPSS/PC (V. 22).

**Table 2:** Salinity and the averages of sedimentological and chemical variables for each core of Edku Lake

Core	Salinity (‰)	S%	Z%	C%	TOC%	TCO_3_%	Cu	Zn	Pb	Cd	Cr	Ni	As
I	3.5	59.7	38.0	2.3	1.59	15.1	47.5	97.1	56.1	2.7	4.0	19.9	4.9
II	3.3	56.7	39.7	3.7	1.75	18.1	37.4	132.5	63.0	4.4	3.5	23.6	8.7
III	3.1	56.3	41.0	2.7	3.35	5.2	43.4	157.2	59.5	6.7	9.9	23.8	24.0
IV	2.5	56.0	40.0	4.0	5.69	5.9	57.7	107.9	92.7	6.8	15.5	36.3	28.9
V	2.8	42.0	52.3	5.7	3.52	5.7	85.6	140.8	93.3	7.4	8.9	21.8	31.1
VI	2.0	40.0	53.7	6.3	4.08	12.9	121.6	151. 8	83.5	7.7	22.3	24.4	34.7
VII	1.8	38.5	50.3	11.3	5.35	14.5	121.7	296.2	82.2	9.6	26.2	40.0	38.6
VIII	1.5	33.0	51.5	15.5	6.30	16.5	148.7	259.6	142.2	10.9	26.6	42.4	43.7
IX	1.3	28.8	54.3	17.0	8.97	19.3	198.0	399.1	145.2	11.4	35.1	48.2	45.3
Min	1.3	28.8	38.0	2.3	1.6	5.2	37.4	97.1	56.1	2.7	3.5	19.9	4.9
Max	3.5	59.7	54.3	17.0	9.0	19.3	198.0	399.1	145.2	11.4	35.1	48.2	45.3
Mean	2.4	45.7	46.7	7.6	4.5	12.6	95.7	193.6	90.8	7.5	16.9	31.2	28.9
Std	0.8	11.6	6.9	5.6	2.3	5.6	55.5	102.1	32.9	2.9	11.2	10.6	14.3

## Results

### Sediment analyses

Sedimentological and geochemical results and pollution assessment were presented briefly in BadrElDin et al. (2022). The following is a summary of the fundamental data used in the present work.

Grain size analyses indicated that the sand fraction was dominating (averages ⁓ 56–60%) the northwest parts of Edku Lake near Boughaz El-Maadia outlet (cores I–III; Table 2). The percent sand decreased eastward, with the muddiest textures in cores VIII and IX (Table 2). There was no trend for the vertical distribution of the grain size classes (sand, silt, and clay). Each core tended to be nearly homogenous regarding sediments grain size (Appendix 1). This was reflected by the absence of sediments stratification in all cores.

Averages of total organic carbon percentages (TOC%) were ranged from ⁓ 1.6% in core I near Boughaz El-Maadia to ⁓9% in core IX, located in the eastern basin near El-Khairy, Edku, and El-Bousily drains (Table 2). Vertically, the total organic carbon showed upward increase in all cores with the lowest percentage in core I (1.58%, 15–25 cm) and the highest of 10.8% in the upper 5 cm in core IX. The total carbonate percentages (TCO_3_%) were varied in average from ⁓ 5% in cores III to ⁓ 19% in core IX (Table 2). The highest total carbonate contents were in concomitant with the presence of molluscan shell fragments (gastropods and bivalves).

**Fig. 2:**
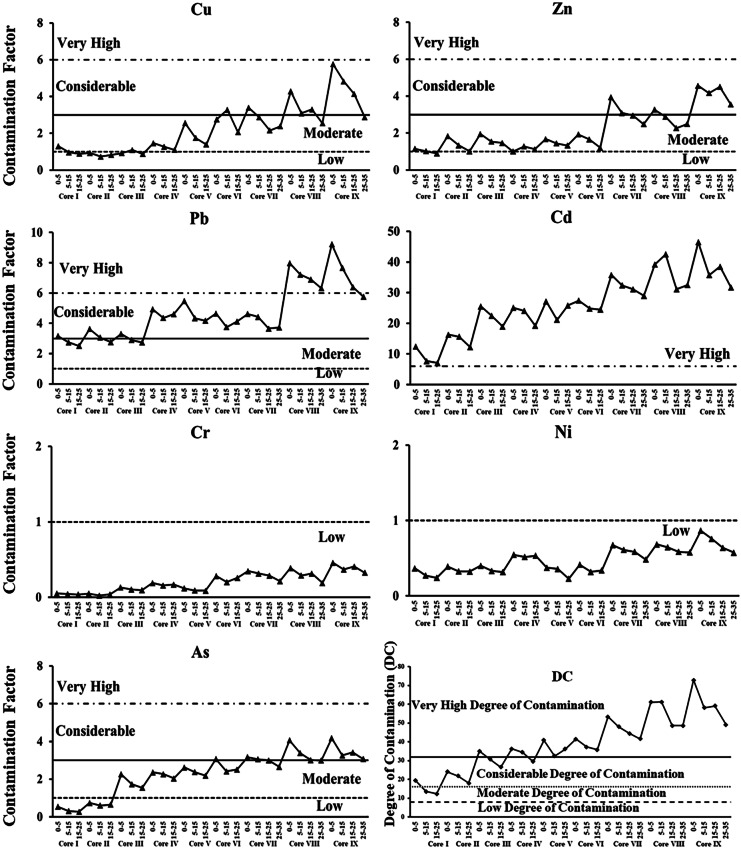
Contamination factor (CF) and degree of contamination (DC) of total metals and in the core sediment of Edku Lake

**Fig. 3:**
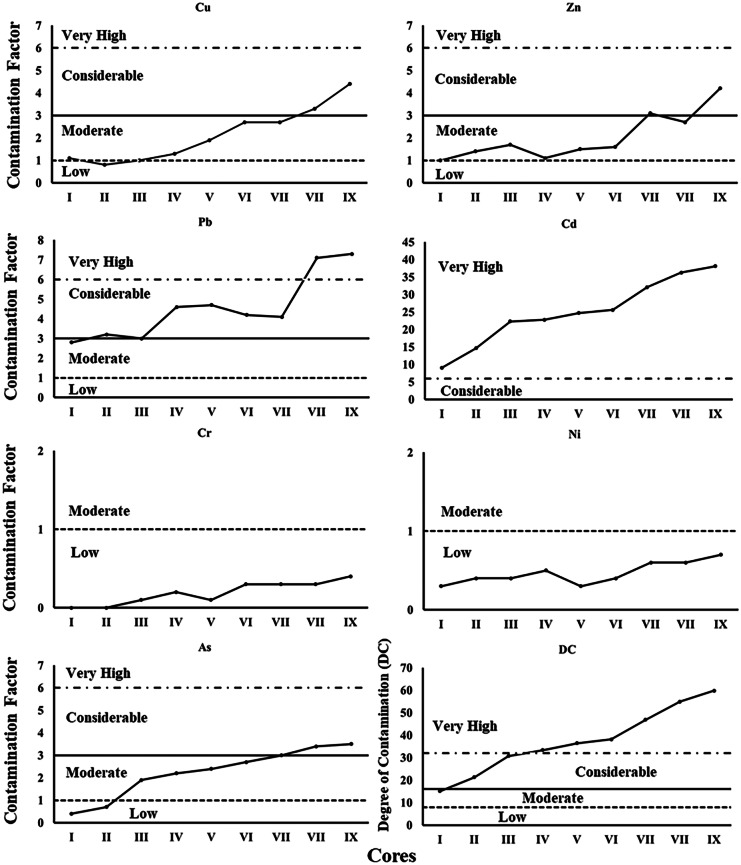
Contamination Factor (CF) and degree of contamination (DC) of total metals for the averages of each core sediment of Edku Lake

The concentrations of the metals in the nine sediment cores at all stations increase generally upward (Appendix 2). Regionally, the highest concentrations were found in cores VII and IX from the eastern part of the lake and in core VIII from the southern part of the lake in the vicinity of Barsik drain (Appendix 2). The CF indicated that sediments (vertically and regionally) have low degrees of contamination with respect to Ni and Cr and a very high degree of contamination with Cd (Table 3, Figs. 2 and 3). The sediments CF for Cu, Zn, and As showed low to considerable degree of contamination where those of Pb had moderate to very high degree of contamination. Regionally, the sediments CF was increased generally eastward. The vertical and regional DC values also showed a high degree of contamination in cores VII–IX (Table 3, Figs. 2 and 3).

**Table 3:** The averages of contamination factor (CF) and degree of contamination (DC) for each core of Edku Lake

Core	Cu	Zn	Pb	Cd	Cr	Ni	Fe	As	DC
I	1.1	1.0	2.8	9.1	0.0	0.3	0.5	0.4	15.1
II	0.8	1.4	3.2	14.7	0.0	0.4	0.2	0.7	21.3
III	1.0	1.7	3.0	22.3	0.1	0.4	0.5	1.9	30.8
IV	1.3	1.1	4.6	22.8	0.2	0.5	0.7	2.2	33.4
V	1.9	1.5	4.7	24.7	0.1	0.3	0.9	2.4	36.5
VI	2.7	1.6	4.2	25.6	0.3	0.4	0.9	2.7	38.2
VII	2.7	3.1	4.1	32.1	0.3	0.6	1.0	3.0	46.9
VII	3.3	2.7	7.1	36.3	0.3	0.6	1.2	3.4	54.9
IX	4.4	4.2	7.3	38.1	0.4	0.7	1.3	3.5	59.8
Min	0.8	1.0	2.8	9.1	0.0	0.3	0.2	0.4	15.1
Max	4.4	4.2	7.3	38.1	0.4	0.7	1.3	3.5	59.8
Mean	2.1	2.0	4.5	25.1	0.2	0.5	0.8	2.2	37.4
Std	1.2	1.1	1.6	9.5	0.1	0.2	0.4	1.1	14.6

**Fig. 4:**
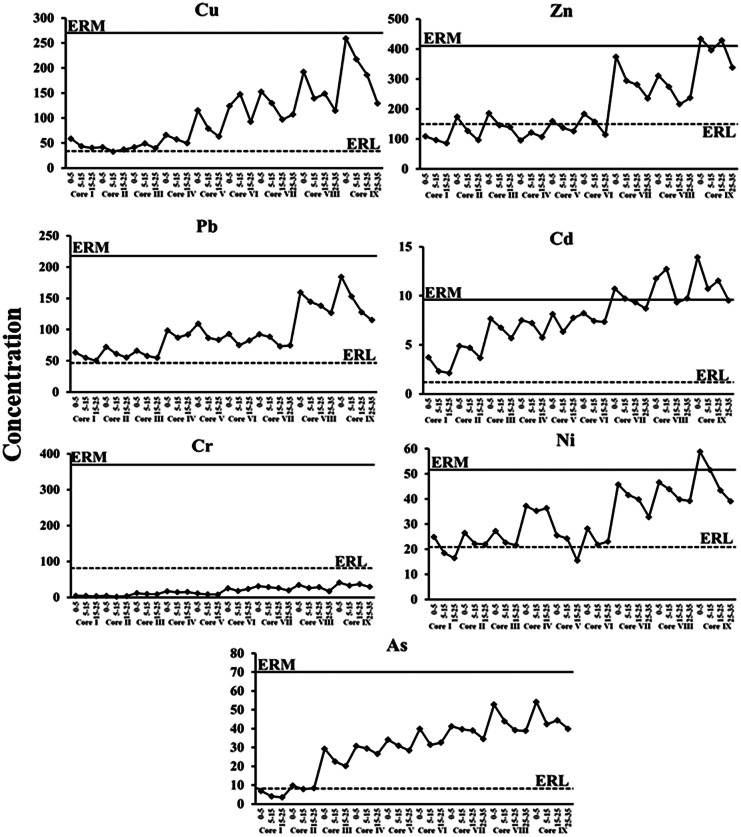
Effects-range low (ERL) and effects-range median (ERM) after Long et al. (1995) in the core sediments of Edku Lake

**Fig. 5:**
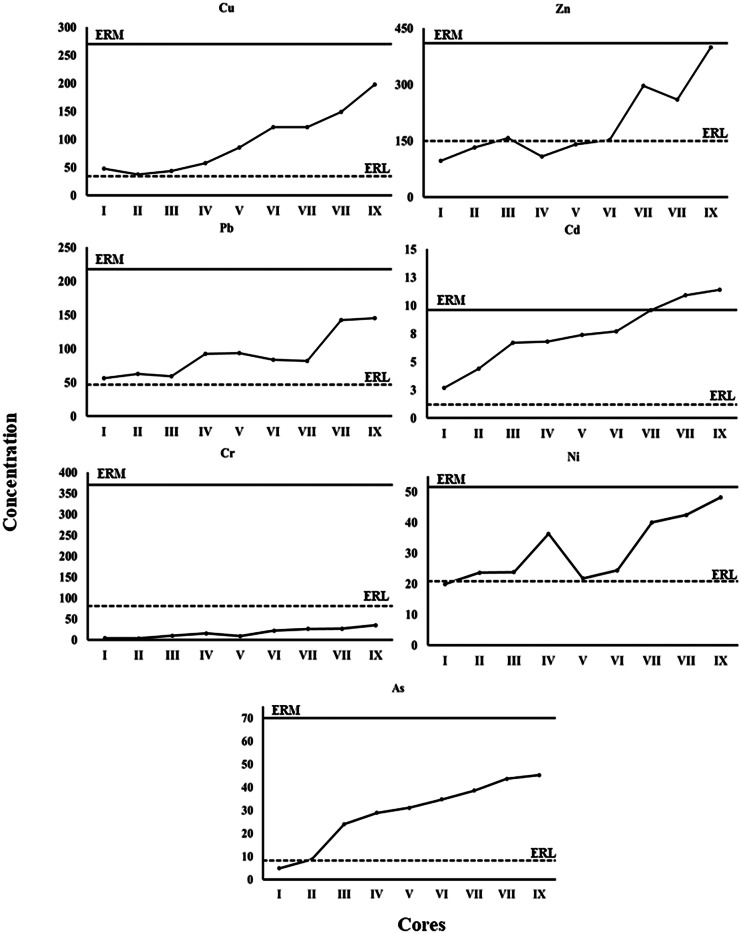
Effects-range low (ERL) and effects-range median (ERM) after Long et al. (1995) for the averages of each core of Edku Lake

To assess sediment quality, we compared the concentrations of the elements assessed to the effects-range low (ERL) and effects-range medium (ERM) guidelines derived from the database of Long et al. (1995) to understand the potential for contamination to affect aquatic organisms. Vertically, Zn and Ni levels in the upper core IX (eastern basin) exceeded the ERM guidelines (Fig. 4). Copper and Pb concentrations at all cores exceeded the ERL threshold but did not reach the ERM concentrations. Only Cr concentrations in all studied cores were below established limits (ERL) for biological effects (Fig. 4). Regionally, Cd concentrations in the core sediments (VII–IX) exceeded the ERM threshold (Fig. 5), indicating potential for frequent detrimental effects.

### Foraminiferal assemblages

**Fig. 6:**
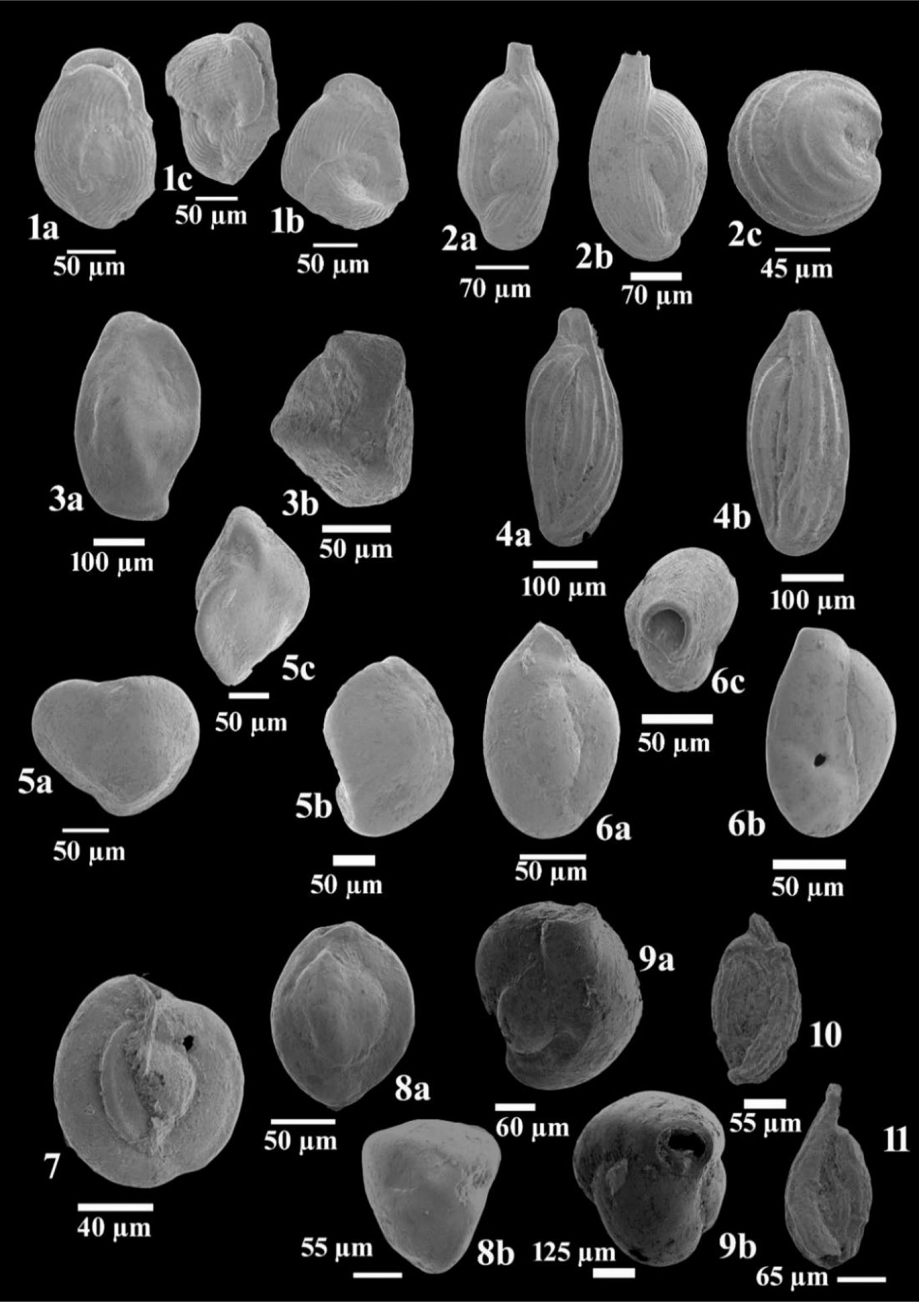
**1** *Vertebralina striata* d'Orbigny: **1a**–**b** side views and **1c** apertural view. **2** *Adelosina mediterranensis* (Le Calvez & Le Calvez): **2a**–**b** side views and **2c** growth stage. **3** *Cycloforina contorta* (d'Orbigny): **3a** side view and **3b** apertural view. **4 ***Massilina paronai* Martinotti: **4a**–**b** side views. **5** *Quinqueloculina auberiana* d'Orbigny: **5a–b** side views and **5c** apertural view. **6** *Quinqueloculina seminula* (Linnaeus): **6a–b** side views and **6c** apertural view. **7** *Miliolinella subrotunda* (Montagu), side view. **8** *Triloculina tricarinata* d'Orbigny: **8a** side view and **8b** apertural view. **9** *Triloculina trigonula* (Lamarck): **9a** side view and **9b** apertural view. **10** *Sigmoilinita costata* Schlumberger, side view. **11** *Sigmoilinita grata* (Terquem), side view

**Fig. 7:**
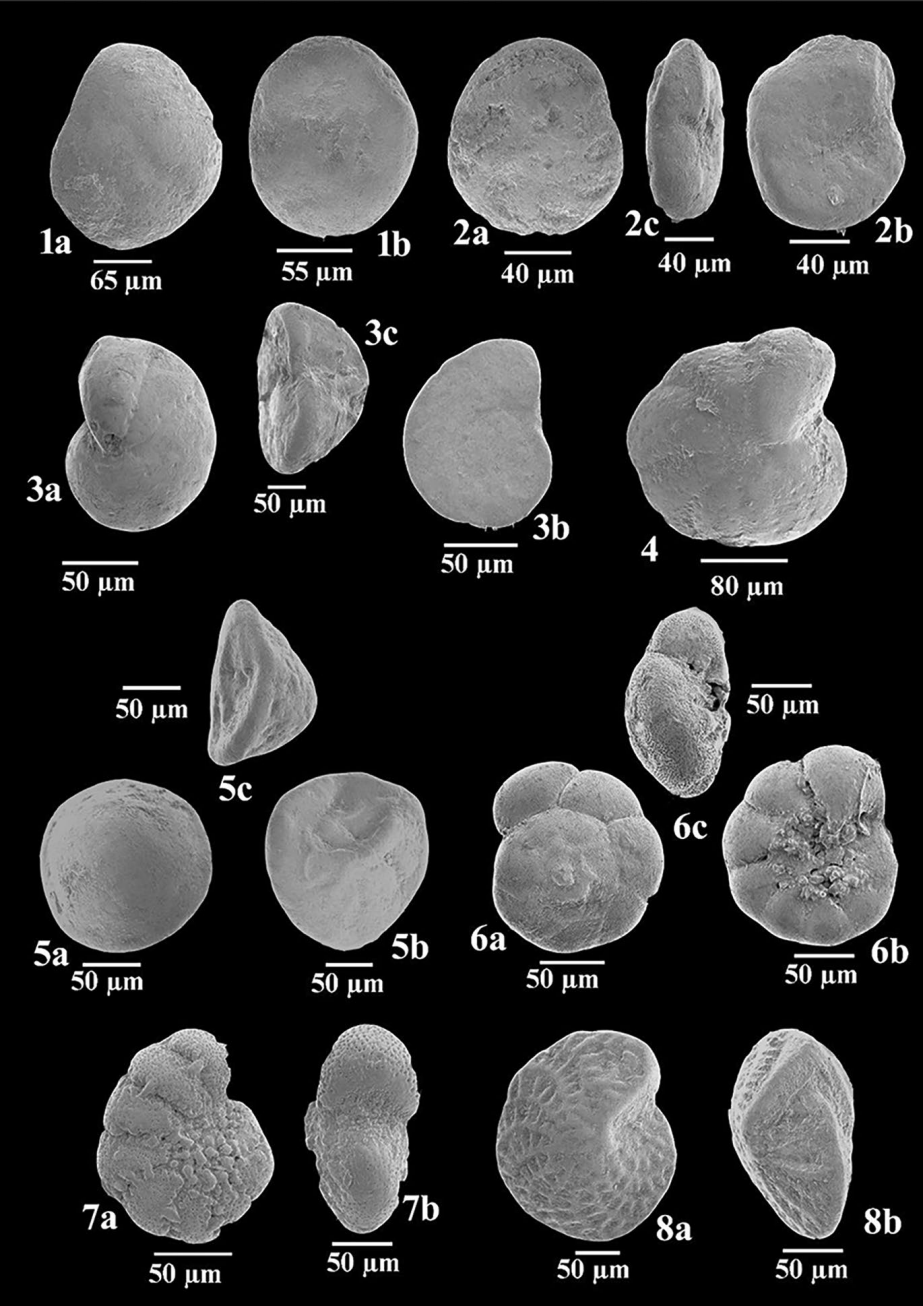
**1 ***Rosalina bradyi* (Cushman): **1a–b** side views. **2 ***Rosalina macropora* (Hofker): **2a**–**b** side views, and **2c** apertural view. **3 ***Cibicides refulgens* Montfort: **3a**–**b** side views, and **3c** apertural view. **4** *Lobatula lobatula* (Walker and Jacob), dorsal view. **5 ***Asterigerinata mamilla* (Williamson): **5a**–**b** side views, and **5c** apertural view. **6** *Ammonia tepida* (Cushman): **6a**–**b** side views, and **6c** apertural view. **7 ***Cribroelphidium excavatum* (Terquem): **7a** side view, and **7b** apertural view. **8 ***Elphidium crispum* (Linnaeus)*,* **8a** side view, and **8b** apertural view

**Fig. 8:**
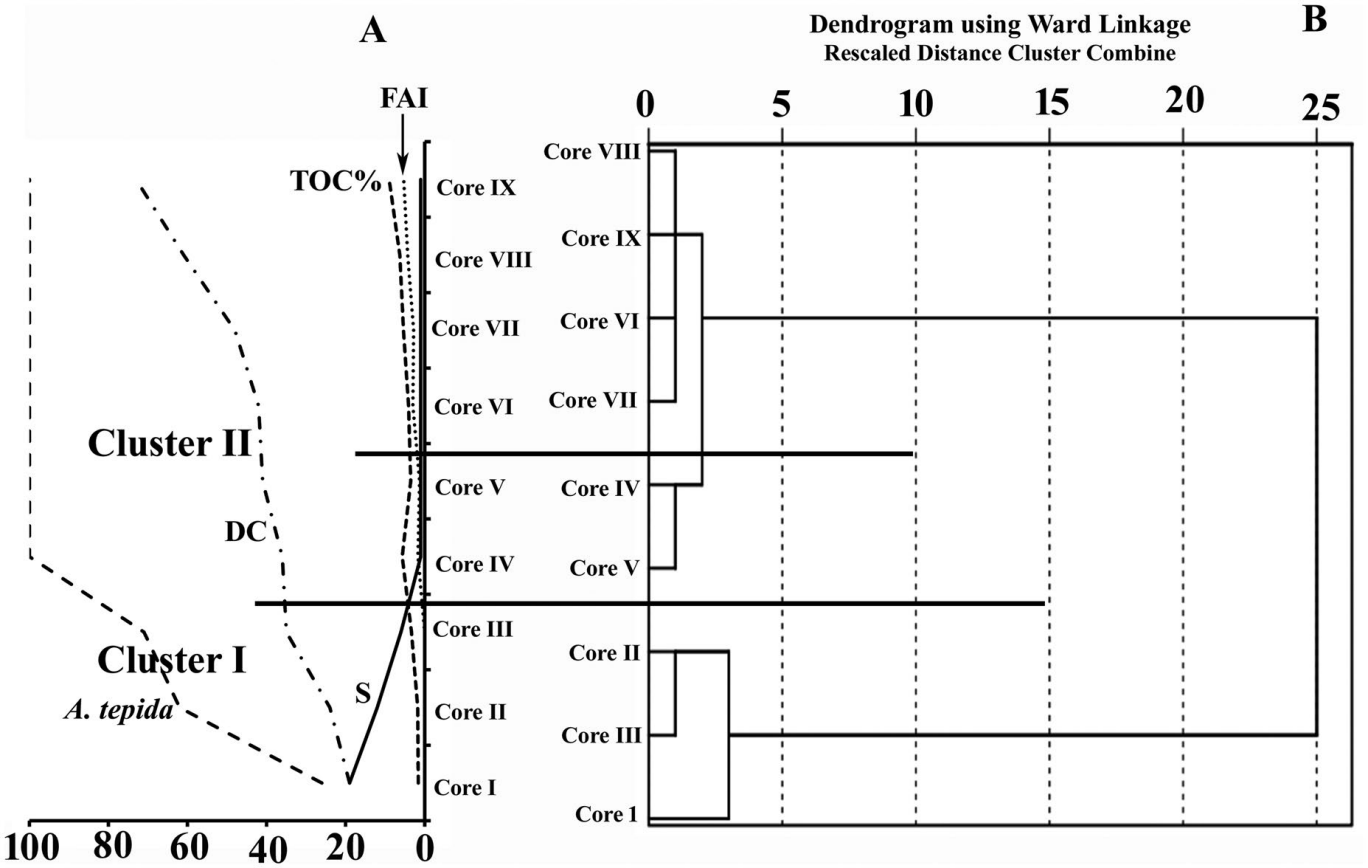
**A** Regional distribution of the averages of species richness (S), *A. tepida* (in percentage), total organic carbon (TOC%), foraminiferal abnormality index (FAI), and degree of contamination (DC) in each core of Edku Lake. **B** Dendrogram of Q-mode cluster analysis using Ward’s linkage method, based on the averages in each core of foraminiferal species abundances and the geo-chemical variables, grouping cores from Edku Lake

Benthic foraminifera were found in all core samples examined (Table 4). From the 24 species identified, thirteen were porcelaneous miliolids, and eleven were hyaline rotaliids (Figs. 6 and 7). Hyaline taxa dominated the assemblages, ranging from 75 to 100% (Table 5). The dominant species were *Ammonia tepida* (84%), *Cribroelphidium excavatum* (3%), and *Quinqueloculina seminula* (⁓ 3%) (Table 5). Porcelaneous foraminifera, including *Q. seminula*, and hyaline *C. excavatum* were only found in the western basin near Boughaz El-Maadia (Table 6). Only tests of *Ammonia tepida* were found in samples from the central and eastern basins. The species richness was much higher in cores I, II, and III from the western side (Tables 5 and 6, Fig. 8A).

**Table 4:** Foraminiferal assemblage composition (%) in the core sediments of Edku Lake

Core	Depth	*Vertebralina striata* d'Orbigny	*Adelosina mediterranensis* (Le Calvez & Le Calvez)	*Spiroloculina antillarum* d'Orbigny	*Cycloforina contorta* (d'Orbigny)	*Massilina paronai* Martinotti	*Quinqueloculina auberiana* d'Orbigny	*Quinqueloculina seminula* (Linnaeus)	*Miliolinella subrotunda* (Montagu)	*Pseudotriloculina rotunda* (d'Orbigny)	*Triloculina tricarinata* d'Orbigny	*Triloculina trigonula* (Lamarck)
I	0–5	0	1	0	2	1	6	2	2	0	1	0
	5–15	3	3	1	3	0	9	1	1	0	0	3
	15–25	2	1	1	1	1	9	0	2	1	1	1
II	0–5	0	3	0	2	0	4	13	1	0	2	0
	5–15	0	2	0	1	0	2	14	0	0	1	0
	15–25	0	1	0	2	0	3	11	0	0	1	0
III	0–5	0	0	0	0	0	0	15	0	0	0	0
	5–15	0	0	0	3	0	0	11	0	0	0	0
	15–25	0	0	0	0	0	0	12	0	0	0	0
IV	0–5	0	0	0	0	0	0	0	0	0	0	0
	5–15	0	0	0	0	0	0	0	0	0	0	0
	15–25	0	0	0	0	0	0	0	0	0	0	0
V	0–5	0	0	0	0	0	0	0	0	0	0	0
	5–15	0	0	0	0	0	0	0	0	0	0	0
	15–25	0	0	0	0	0	0	0	0	0	0	0
VI	0–5	0	0	0	0	0	0	0	0	0	0	0
	5–15	0	0	0	0	0	0	0	0	0	0	0
	15–25	0	0	0	0	0	0	0	0	0	0	0
VII	0–5	0	0	0	0	0	0	0	0	0	0	0
	5–15	0	0	0	0	0	0	0	0	0	0	0
	15–25	0	0	0	0	0	0	0	0	0	0	0
	25–35	0	0	0	0	0	0	0	0	0	0	0
VIII	0–5	0	0	0	0	0	0	0	0	0	0	0
	5–15	0	0	0	0	0	0	0	0	0	0	0
	15–25	0	0	0	0	0	0	0	0	0	0	0
	25–35	0	0	0	0	0	0	0	0	0	0	0
IX	0–5	0	0	0	0	0	0	0	0	0	0	0
	5–15	0	0	0	0	0	0	0	0	0	0	0
	15–25	0	0	0	0	0	0	0	0	0	0	0
	25–35	0	0	0	0	0	0	0	0	0	0	0

Core	Depth	*Sigmoilinita costata* **Schlumberger**	*Sigmoilinita grata* (*Terquem*)	*Rosalina bradyi* (Cushman)	*Rosalina macropora* (Hofker)	*Cibicides refulgens* Montfort	*Lobatula lobatula* (Walker & Jacob)	*Asterigerinata mamilla* (Williamson)	*Ammonia beccarii* (Linnaeus)	*Ammonia parkinsoniana* (d'Orbigny)	*Ammonia tepida* (Cushman)	*Cribroelphidium excavatum* (*Terquem*)	*Elphidium crispum* (*Linnaeus*)	*Elphidium macellum* (Fichtel & Moll)
I	0–5	1	1	2	2	13	1	5	0	7	36	12	3	2
	5–15	1	0	0	1	8	4	7	2	7	29	13	3	1
	15–25	0	0	3	1	13	3	9	3	4	27	8	5	4
II	0–5	0	0	2	0	1	0	1	1	0	62	8	0	0
	5–15	1	0	0	2	1	0	1	7	3	54	9	0	2
	15–25	1	0	1	2	1	0	4	2	1	55	13	0	2
III	0–5	0	0	0	0	2	0	3	1	0	71	8	0	0
	5–15	0	0	0	0	3	0	3	4	0	71	5	0	0
	15–25	0	0	1	0	1	0	2	3	0	75	6	0	0
IV	0–5	0	0	0	0	0	0	0	0	0	100	0	0	0
	5–15	0	0	0	0	0	0	0	0	0	100	0	0	0
	15–25	0	0	0	0	0	0	0	0	0	100	0	0	0
V	0–5	0	0	0	0	0	0	0	0	0	100	0	0	0
	5–15	0	0	0	0	0	0	0	0	0	100	0	0	0
	15–25	0	0	0	0	0	0	0	0	0	100	0	0	0
VI	0–5	0	0	0	0	0	0	0	0	0	100	0	0	0
	5–15	0	0	0	0	0	0	0	0	0	100	0	0	0
	15–25	0	0	0	0	0	0	0	0	0	100	0	0	0
VII	0–5	0	0	0	0	0	0	0	0	0	100	0	0	0
	5–15	0	0	0	0	0	0	0	0	0	100	0	0	0
	15–25	0	0	0	0	0	0	0	0	0	100	0	0	0
	25–35	0	0	0	0	0	0	0	0	0	100	0	0	0
VIII	0–5	0	0	0	0	0	0	0	0	0	100	0	0	0
	5–15	0	0	0	0	0	0	0	0	0	100	0	0	0
	15–25	0	0	0	0	0	0	0	0	0	100	0	0	0
	25–35	0	0	0	0	0	0	0	0	0	100	0	0	0
IX	0–5	0	0	0	0	0	0	0	0	0	100	0	0	0
	5–15	0	0	0	0	0	0	0	0	0	100	0	0	0
	15–25	0	0	0	0	0	0	0	0	0	100	0	0	0
	25–35	0	0	0	0	0	0	0	0	0	100	0	0	0

**Table 5:** Miliolida%, Rotaliida%, species richness (S), degrees of deformation groups (A, B, and C) of *Ammonia tepida*, and foraminiferal abnormality index (FAI) in the core sediments of Edku Lake

Core	Depth	Miliolida%	Rotaliida%	Species richness (S)	Group A	Group B	Group C	FAI
I	0–5	17	83	19	ND	ND	ND	0
	5–15	25	75	19	ND	ND	ND	0
	15–25	20	80	21	ND	ND	ND	0
II	0–5	25	75	12	ND	ND	ND	0
	5–15	21	79	14	ND	ND	ND	0
	15–25	19	81	15	ND	ND	ND	0
III	0–5	15	85	6	ND	ND	ND	0
	5–15	14	86	7	ND	ND	ND	0
	15–25	12	88	7	ND	ND	ND	0
IV	0–5	0	100	1	1	1	ND	2
	5–15	0	100	1	2	1	ND	3
	15–25	0	100	1	ND	ND	ND	0
V	0–5	0	100	1	2	2	ND	4
	5–15	0	100	1	ND	ND	ND	0
	15–25	0	100	1	ND	ND	ND	0
VI	0–5	0	100	1	4	1	ND	5
	5–15	0	100	1	2	1	ND	3
	15–25	0	100	1	1	ND	ND	1
VII	0–5	0	100	1	1	3	3	7
	5–15	0	100	1	ND	3	ND	3
	15–25	0	100	1	1	ND	ND	1
	25–35	0	100	1	ND	ND	ND	0
VIII	0–5	0	100	1	ND	5	3	8
	5–15	0	100	1	ND	4	1	5
	15–25	0	100	1	1	2	ND	3
	25–35	0	100	1	1	ND	ND	1
IX	0–5	0	100	1	ND	3	7	10
	5–15	0	100	1	ND	3	3	6
	15–25	0	100	1	ND	4	ND	4
	25–35	0	100	1	1	ND	ND	1

*ND* not detected

**Table 6:** The relative abundance (≥ 5% in at least one core) and foraminiferal indices for each core of Edku Lake

Core	*Quinqueloculina auberiana* d'Orbigny	*Quinqueloculina seminula* (Linnaeus)	*Cibicides refulgens* Montfort	*Asterigerinata mamilla* (Williamson)	*Ammonia parkinsoniana* (d'Orbigny)	*Ammonia tepida* (Cushman)	*Cribroelphidium excavatum* (*Terquem*)	Miliolida%	Rotaliida%	Species Richness	FAI
I	8.0	1.0	11.3	7.0	6.0	30.7	11.0	20.7	79.3	20	0.0
II	3.0	12.7	1.0	2.0	1.3	57.0	10.0	21.7	78.3	14	0.0
III	0.0	12.7	2.0	2.7	0.0	72.3	6.3	13.7	86.3	7	0.0
IV	0.0	0.0	0.0	0.0	0.0	100	0.0	0.0	100.0	1	1.7
V	0.0	0.0	0.0	0.0	0.0	100	0.0	0.0	100.0	1	1.3
VI	0.0	0.0	0.0	0.0	0.0	100	0.0	0.0	100.0	1	3.0
VII	0.0	0.0	0.0	0.0	0.0	100	0.0	0.0	100.0	1	2.8
VIII	0.0	0.0	0.0	0.0	0.0	100	0.0	0.0	100.0	1	4.3
IX	0.0	0.0	0.0	0.0	0.0	100	0.0	0.0	100.0	1	5.3
Min	0.0	0.0	0.0	0.0	0.0	30.7	0.0	0.0	78.3	1	0.0
Max	8.0	12.7	11.3	7.0	6.0	100.0	11.0	21.7	100.0	20	5.3
Mean	1.2	2.9	1.6	1.3	0.8	84.4	3.0	6.2	93.8	5	2.0
Std	2.7	5.5	3.7	2.4	2.0	25.6	4.7	9.6	9.6	7	1.9

Morphological abnormalities, evaluated using the foraminiferal abnormality index (FAI), ranged between 0 and 10% (average for each core 0–5%) and were only found in specimens of *A. tepida* (Table 5, Appendices 3, 4, 5, and 6). No aberrant specimens were found in western cores I–III, whereas the FAI increased gradually eastward in the central and eastern basins, reaching its maximum value in the upper 5 cm in core IX (10%, Tables 5 and 6). The percentages of deformed specimens decreased downcore in cores V–IX.

*Ammonia tepida* specimens showed a wide range of deformation. The recorded morphological abnormalities included aberrant chamber shape, abnormal chamber size, additional chambers, abnormal test growth, elongated axes of rotation, spiroconvex coiling, twisted test, excess deposition of calcium carbonate, irregular periphery, and complex deformities. We classified the degree of test deformation according to the following criteria: (1) mild deformation (group A, Appendix 3), where the test maintains the general characteristics of a normal test and could be identified easily; (2) moderate deformation (group B, Appendices 4 and 5), where tests exhibited more than one type of deformation, yet the test retains the general characteristics of normal tests; and (3) extreme deformation (group C, Appendix 6), where the tests show multiple complex deformations, such that species identification was very difficult in some instances. The degree of deformation increased toward the eastern basin of Edku Lake (Table 5, Fig. 8A). Abnormal specimens of groups A and B were represented in cores of the central and eastern lake basins (cores IV**–**IX), whereas the specimens belonging to group C were found only in cores VII**–**IX. The spatial and vertical distributions of malformed specimens were consistent with contaminant concentrations.

### Multivariate analyses

One-way ANOVA revealed that the foraminiferal assemblages and the determined variables were significantly different among the cores (*f* = 3.1; *p* = 0.003). A dendrogram generated by hierarchical Q-mode cluster analysis showed two groups of samples that reflected the regional distribution of cores (Fig. 8B). Cluster I included cores I, II, and III from the northeastern basin near Boughaz El-Maadia outlet, whereas cluster II grouped the central cores (IV–V) and the eastern cores (VI–IX) close to the major drains (El-Khairy, Edku, El-Bousily, and Barsik) (Figs. 1 and 8B).

**Fig. 9:**
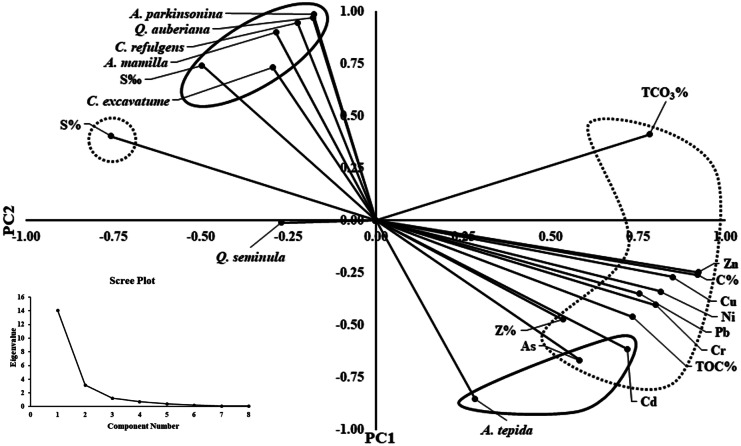
Principal components analysis (PCA) for the average values for foraminiferal species abundance and the chemical and sedimentological variables for each core from Edku Lake. In frame the scree plot representing the number of components

The principal component analysis (PCA) was used to realize the environmental variables probably affecting the distribution of foraminiferal assemblages. The PCA revealed that the first two components clarify ~ 86.3% of the total data variance (Fig. 9, Appendix 7). The heavy metals, sand, silt, clay, TOC, and TCO_3_ are the predominant variables in the first component, whereas the major contributors to the second component are the foraminiferal species, salinity, Cd, and As. *Ammonia tepida* showed high abundances positively related to the lower salinities and the higher concentrations of Cd and As. The foraminiferal species, *Ammonia parkinsoniana*, *Quinqueloculina auberiana*, *Cibicides refulgens*, *Asterigerinata mamilla*, and *Cribroelphidium excavatum*, showed a preference for areas with marine influence and lower Cd and As concentrations.

## Discussion

### Sediment analyses

The sedimentological and geochemical results and sediment quality assessment were briefly discussed in BadrElDin et al. (2022). Grain size analyses revealed that the lake sediments become finer in the eastward direction. Sediment texture in core I from the northwest side of the lake near Boughaz El-Maadia outlet is sandy, where water and sediments exchange between Lake Edku and Abu Qir Bay take place. The regional distribution of mud% (Z% + C%) was ranged from 40.3 to 71.3% with an average of 54.3%. Abdallah and Morsy (2013) stated that percent mud ranged from 11 to 77% with an average of 47%.

Regionally, the total organic carbon generally increased in the eastward direction (range: ⁓ 1.6–9%) reaching its maximum near El-Khairy, Edku, and El-Bousily drains. Organic matter in the sediment originates both from in situ decomposition of plant and animal matter by bacteria and from the drains, especially in the eastern basin due to industrial and agricultural activities along this part of the lake (Masoud et al., 2005). Total carbonate percentages (TCO_3_%) were generally low in Lake Edku core sediments both regionally (range: 5–19%) and vertically (range: 4–21%) with obvious percentage increase in concordance with the occurrence of molluscan shell fragments. Carbonates in lake sediments were mostly bioclastics consisting of fragments of molluscan shells, ostracods, benthic foraminifera, and other calcareous organisms (Abdallah & Morsy, 2013).

The highest Cu, Pb, Zn, Cd, Cr, Ni, and As concentrations were found in cores VII and IX, from the eastern part of the lake affected by Edku and El-Khairy drains, and in core VIII in the southern part of the lake in the vicinity of Barsik drain. These concentrations can be attributed to discharges from surrounding agriculture and domestic drains. Moreover, Cd and Pb are the major pollutants in the lake sediments, with highest concentrations found in cores VIII and IX. Roberts (2014) noted that because Cd is known to be linked with phosphatic fertilizers, Cd pollution can be associated with runoff from agricultural lands. The contamination factors and degree of contamination herein indicate that chronic anthropogenic pollution is likely the major factor controlling the distribution of metal contaminants in the lake sediments.

### Foraminiferal assemblages

The foraminiferal assemblage in Lake Edku is very sparse, likely in response to multiple levels of environmental stress including freshwater influence, high organic carbon load, and elevated concentrations of Cd and Pb particularly in the eastern basin near El-Khairy and Barsik drains. The foraminiferal assemblage is dominated by two hyaline species, *A. tepida* and *C. excavatum*, and one porcelaneous species, *Q. seminula*. All three have been recognized as stress tolerant in previous studies (e.g., Debenay, 2009; Eichler et al., 2007; Martins et al., 2013). Recently *C. excavatum* was categorized as (third order) opportunistic species (Jorissen et al., 2018; Martínez-Colón et al., 2018). In this work, *C. excavatum* showed a significant negative correlation with Cd and *A. tepida* (Fig. 9). *Cribroelphidium excavatum*, the third-order opportunistic species, was abundant in the northwest parts of Edku Lake distant from areas of highly polluted conditions (Jorissen et al., 2018). Miliolids were only found in the western basin in sandy sediment and lower pollution condition. Generally, miliolids favor the sandy substrate (Elshanawany et al., 2011; Li et al., 2015) and are sensitive to heavy metals pollution (Samir & El-Din, 2001). Some miliolids such as *Q. seminula* showed no response to heavy metals pollution (Martins et al., 2013). This could explain the dominance of *Q. seminula* in the northwest parts of Edku Lake near Boughaz El-Maadia outlet. The highest species richness (24 species total) was found in core I near Boughaz El-Maadia, where salinity is highest, and the concentrations of all potentially toxic elements assessed are the lowest. Only the most stress-tolerant specie, *A. tepida*, was even found in the central and eastern lake basins, which are characterized by low salinity, higher percentages of organic matter, and higher concentrations of all the elements analyzed.

Test deformation was only observed in *A. tepida*, which was the only species found in the central and eastern basins. The most deformed specimens belonging to moderate and extreme deformation degrees (group B and group C) were found in cores VII–IX. In addition, the vertical distribution of FAI and degree of deformation decreased downcore, consistent with decreasing metals concentrations and their pollution indices. Foraminiferal abnormality index (FAI) increased with decreasing salinity and with increasing organic carbon and heavy metal concentrations, consistent with the work of Morvan et al. (2004), who found that FAI exceeding 1% reflected contaminated environments. Several studies reported that BF living in hyposalinity waters or lagoons characterized by fluctuations of salinity tend to become deformed (Boltovskoy et al., 1991). In this work, correlating FAI to total (bulk) heavy metals concentrations may be a quite problematic issue. Several studies used the bioavailable fraction (releasable) of the heavy metals (e. g., Martínez-Colón et al., 2017, 2018, Raposo et al., 2022). However, the total (bulk) heavy metals concentrations could be effective for biomonitoring paralic coastal environments. The high contents of TOC particularly in the eastern cores (VII–IX) and the significant positive correlation observed with total heavy metal concentrations (Fig. 9) clarify that OC could play an essential role in bioavailability of heavy metals for bottom biota (BadrElDin et al., 2022; Liang et al., 2019). On the other hand, the significant positive correlations between heavy metals and muds (Z% and C%; Fig. 9) indicated that muds could adsorb and provide a sink for the heavy metals (Martínez-Colón et al., 2017). Minor changes in salinity or pH could lead to bioavailability of heavy metals to foraminifers due to desorption or scavenging from mud-sized sediment surfaces (Martínez-Colón et al., 2009, 2018). Accordingly, the *A. tepida* test deformities in Edku Lake sediments could be attributed to multiple stressors including the low salinity and the high contents of heavy metals.

According to Murray (2014), the fluctuations in salinity are a major ecological stressor for foraminiferal richness and distribution. Miliolids were only recorded near Boughaz ElMadiaa, in association with salinity closer to normal marine. Paralic environments of the Nile Delta (Manzalla, Burullus, and Edku lakes) showed similar results (Badr-ElDin et al., 2019; Elshanawany et al., 2019; Orabi et al., 2017). The species *A. tepida*, *C. excavatum*, and *Q. seminula* are cosmopolitan euryhaline species (e.g., Debenay, 2009; Eichler et al., 2007; Martins et al., 2013), though the latter two are less tolerant to hyposalinity than *A. tepida* (El Baz, 2017). Thus, foraminiferal distributions in Lake Edku are certainly controlled by the salinity gradient.

Another source of stress is related to abundant organic carbon associated with muddy sediments, particularly in central and eastern basins. *Ammonia tepida* is highly tolerant of numerous types of pollution, including fertilizers, municipal sewage, and hydrocarbons (e.g., Debenay et al., 2006; Frontalini et al., 2010; Martins et al., 2019), as well as to high percentages of organic carbon (Badr-ElDin et al., 2019; Melis et al., 2019). Similarly, *C. excavatum* and *Q. seminula* have been reported in sediments enriched in organic carbon (e.g., Eichler et al., 2007; Mangoni et al., 2016). However, we found only *A. tepida* in the organic-rich sediments in the polluted central and eastern basins of the lake, supporting the argument that salinity is the major factor controlling foraminiferal taxa.

The eastern basin cores exhibited the highest concentrations of the evaluated elements. Contamination factors (Fig. 2) indicated that sediments from cores V–IX exhibited very high degrees of contamination, particularly with Cd and Pb. The effects range categories (Fig. 4) indicated that concentrations of all evaluated elements except Zn and Cr exceeded the ERL threshold in most cores. Concentrations of Cd exceeded the ERM threshold in cores VII–IX, while Zn and Ni concentrations exceeded such thresholds only in core IX. Caruso et al. (2011) reported that *A. tepida* can tolerate particularly high concentrations of heavy metals, and in the eastern basin of Edku Lake, *A. tepida* reacted to the high concentrations of heavy metals by the gradual increase in the number of deformed specimens and complexity of test distortion. Thus, the high values of some or all of these elements likely produced deleterious effects documented by the eastward increase in numbers of deformed specimens and the increase in degree of deformation. In nearly all indices, contamination and potential for effects increased up-core, especially in the eastern basin sites. Moreover, Zn concentrations in core I and in the subsurface samples from cores II to V were below the ERL threshold, increased dramatically in the surface of core VII and even exceeded the ERM threshold in the core IX (Fig. 4). Our results were consistent with those reported by Orabi et al. (2017), who found that deformation became more severe with increasing Cd and Zn concentrations. Thus, while the salinity gradient can explain why only *A. tepida* was found in the central and eastern basins of Lake Edku, elevated concentrations of Cd and Pb, and possibly of Zn and other potentially toxic elements, very likely are responsible for the increase in deformed *A. tepida* tests observed in proximity to drains discharging polluted waters into the central and eastern basins.

## Conclusions

The west to east decline in salinity appeared to be the primary factor limiting foraminiferal distributions at Lake Edku. Tests of the species *A. tepida* were found in sediments from all cores, whereas tests of *Q. seminula*, *C. excavatum*, and 21 other species were found only in cores from the western basin, which receives seawater influx from the Mediterranean Sea. Higher organic carbon and heavy-metal concentrations in Lake Edku sediments reflect pollutants associated with agricultural and urban activities. Concentrations of organic carbon and most of the heavy elements increased eastward, peaking in the surface sample nearest the major drain discharge. Elements found in concentrations with the highest potential for affecting aquatic organisms were cadmium and lead and to a lesser extent zinc and nickel. Foraminiferal assemblages are responding to the multiple ecological stressors as indicated by the west to east decrease in species richness and by the increase in and degree of morphological deformation expressed. Benthic foraminifera are particularly suitable as environmental proxies and should be used for periodic monitoring of environmental quality of paralic coastal environments, such as those of the Nile Delta that are influenced by multiple natural and anthropogenic environmental sources of stress.

## Supplementary Information

Below is the link to the electronic supplementary material.

Supplementary file1 (DOCX 2167 kb)

## Data Availability

All data generated or analyzed during this study are included in this published article and its Appendix information file.
