# Supplementary material for: Biomonitoring of a Nile Delta Lake using benthic foraminifera
Source: Environ Monit Assess. 2022 Nov 7;195(1):79. doi: 10.1007/s10661-022-10611-w (PMC9640433; doi:10.1007/s10661-022-10611-w)
Supplement: Supplementary file 1 — Supplementary file1 (DOCX 2167 kb) [file 10661_2022_10611_MOESM1_ESM.docx]

Appendix 1. Cores’ locations and salinity, sand (S%), silt (Z%), clay (C%), total carbonates (TCO_3_%), and total organic carbon (TOC%) percentages in the core sediments of Edku Lake (after BadrElDin et al., 2022).

| Core | Latitudes | Longitudes | Depth | S% | Z% | C% | TOC% | TCO_3_% |
| --- | --- | --- | --- | --- | --- | --- | --- | --- |
| I | 31°15ʹ56ʺ | 30°10ʹ94ʺ | 0-5 | 60 | 37 | 3 | 1.60 | 9.35 |
|  |  |  | 5-15 | 59 | 39 | 2 | 1.59 | 17.67 |
|  |  |  | 15-25 | 60 | 38 | 2 | 1.58 | 18.24 |
| II | 31°15ʹ17ʺ | 30°10ʹ34ʺ | 0-5 | 57 | 38 | 5 | 1.86 | 19.26 |
|  |  |  | 5-15 | 58 | 40 | 2 | 1.79 | 17.15 |
|  |  |  | 15-25 | 55 | 41 | 4 | 1.60 | 18.01 |
| III | 31°15ʹ36ʺ | 30°11ʹ35ʺ | 0-5 | 55 | 42 | 3 | 3.64 | 6.35 |
|  |  |  | 5-15 | 58 | 40 | 2 | 3.26 | 4.13 |
|  |  |  | 15-25 | 56 | 41 | 3 | 3.14 | 5.23 |
| IV | 31°14ʹ38ʺ | 30°11ʹ18ʺ | 0-5 | 54 | 41 | 5 | 6.57 | 7.26 |
|  |  |  | 5-15 | 58 | 39 | 3 | 5.62 | 5.44 |
|  |  |  | 15-25 | 56 | 40 | 4 | 4.89 | 4.98 |
| V | 31°15ʹ28ʺ | 30°12ʹ53ʺ | 0-5 | 42 | 50 | 8 | 3.34 | 4.23 |
|  |  |  | 5-15 | 39 | 56 | 5 | 4.88 | 7.93 |
|  |  |  | 15-25 | 45 | 51 | 4 | 2.34 | 4.91 |
| VI | 31°14ʹ32ʺ | 30°12ʹ10ʺ | 0-5 | 36 | 55 | 9 | 5.06 | 14.15 |
|  |  |  | 5-15 | 47 | 49 | 4 | 3.03 | 12.12 |
|  |  |  | 15-25 | 37 | 57 | 6 | 4.15 | 12.46 |
| VII | 31°15ʹ26ʺ | 30°13ʹ38ʺ | 0-5 | 38 | 45 | 17 | 6.28 | 13.37 |
|  |  |  | 5-15 | 42 | 49 | 9 | 5.12 | 12.15 |
|  |  |  | 15-25 | 41 | 51 | 8 | 4.42 | 15.39 |
|  |  |  | 25-35 | 33 | 56 | 11 | 5.59 | 16.87 |
| VIII | 31°14ʹ18ʺ | 30°13ʹ28ʺ | 0-5 | 28 | 53 | 19 | 7.68 | 20.82 |
|  |  |  | 5-15 | 34 | 52 | 14 | 6.61 | 17.85 |
|  |  |  | 15-25 | 39 | 50 | 11 | 7.46 | 15.64 |
|  |  |  | 25-35 | 31 | 51 | 18 | 3.46 | 11.52 |
| IX | 31°14ʹ57ʺ | 30°14ʹ13ʺ | 0-5 | 27 | 54 | 19 | 10.80 | 15.45 |
|  |  |  | 5-15 | 31 | 53 | 16 | 8.03 | 21.10 |
|  |  |  | 15-25 | 28 | 54 | 18 | 9.44 | 21.28 |
|  |  |  | 25-35 | 29 | 56 | 15 | 7.60 | 19.54 |

Appendix 2. Heavy metals concentrations in the core sediments of Edku Lake (after BadrElDin et al., 2022).

| Core | Depth | Cu ppm | Zn ppm | Pb ppm | Cd ppm | Cr ppm | Ni ppm | As ppm |
| --- | --- | --- | --- | --- | --- | --- | --- | --- |
| I | 0-5 | 58.83 | 109.04 | 63.27 | 3.73 | 4.71 | 24.89 | 6.99 |
|  | 5-15 | 43.44 | 96.61 | 54.87 | 2.31 | 3.93 | 18.48 | 4.10 |
|  | 15-25 | 40.22 | 85.54 | 50.28 | 2.12 | 3.44 | 16.44 | 3.56 |
| II | 0-5 | 41.96 | 174.15 | 72.44 | 4.89 | 4.57 | 26.52 | 9.71 |
|  | 5-15 | 33.06 | 126.99 | 61.21 | 4.69 | 2.21 | 22.20 | 7.88 |
|  | 15-25 | 37.04 | 96.25 | 55.35 | 3.68 | 3.65 | 21.97 | 8.48 |
| III | 0-5 | 41.60 | 185.67 | 66.11 | 7.66 | 11.86 | 27.28 | 29.38 |
|  | 5-15 | 49.07 | 146.29 | 57.85 | 6.74 | 9.36 | 22.64 | 22.52 |
|  | 15-25 | 39.55 | 139.54 | 54.67 | 5.69 | 8.55 | 21.57 | 20.15 |
| IV | 0-5 | 66.10 | 95.13 | 98.58 | 7.52 | 17.07 | 37.20 | 30.73 |
|  | 5-15 | 57.29 | 121.26 | 87.19 | 7.22 | 14.14 | 35.24 | 29.54 |
|  | 15-25 | 49.65 | 107.24 | 92.25 | 5.76 | 15.21 | 36.34 | 26.54 |
| V | 0-5 | 115.31 | 159.17 | 109.48 | 8.15 | 10.64 | 25.53 | 34.13 |
|  | 5-15 | 78.79 | 137.19 | 86.82 | 6.34 | 8.15 | 24.30 | 30.90 |
|  | 15-25 | 62.83 | 126.17 | 83.52 | 7.75 | 7.93 | 15.48 | 28.39 |
| VI | 0-5 | 124.37 | 183.40 | 92.98 | 8.23 | 25.54 | 28.29 | 39.90 |
|  | 5-15 | 147.79 | 157.67 | 75.03 | 7.44 | 18.18 | 21.81 | 31.42 |
|  | 15-25 | 92.53 | 114.27 | 82.56 | 7.34 | 23.29 | 23.08 | 32.65 |
| VII | 0-5 | 152.90 | 374.20 | 92.34 | 10.74 | 31.29 | 45.81 | 41.24 |
|  | 5-15 | 129.64 | 293.64 | 88.47 | 9.72 | 28.38 | 41.60 | 39.71 |
|  | 15-25 | 96.94 | 281.27 | 73.21 | 9.33 | 25.93 | 39.85 | 38.98 |
|  | 25-35 | 107.33 | 235.50 | 74.62 | 8.70 | 19.36 | 32.81 | 34.57 |
| VIII | 0-5 | 192.62 | 311.17 | 159.49 | 11.75 | 34.93 | 46.58 | 52.83 |
|  | 5-15 | 139.15 | 274.38 | 144.57 | 12.75 | 26.09 | 43.91 | 43.97 |
|  | 15-25 | 148.60 | 215.89 | 138.07 | 9.34 | 28.36 | 39.83 | 39.20 |
|  | 25-35 | 114.57 | 237.06 | 126.56 | 9.74 | 17.14 | 39.25 | 38.88 |
| IX | 0-5 | 259.53 | 433.89 | 184.40 | 13.94 | 41.21 | 58.89 | 54.25 |
|  | 5-15 | 217.31 | 396.18 | 153.18 | 10.72 | 33.14 | 51.54 | 42.37 |
|  | 15-25 | 186.28 | 428.31 | 127.87 | 11.56 | 36.86 | 43.43 | 44.44 |
|  | 25-35 | 129.05 | 337.98 | 115.20 | 9.52 | 29.36 | 39.06 | 39.95 |
| Background Values^*^ | | 45 | 95 | 20 | 0.3 | 90 | 68 | 13 |

* Background Values after Turekian and Wedepohl (1961)

**
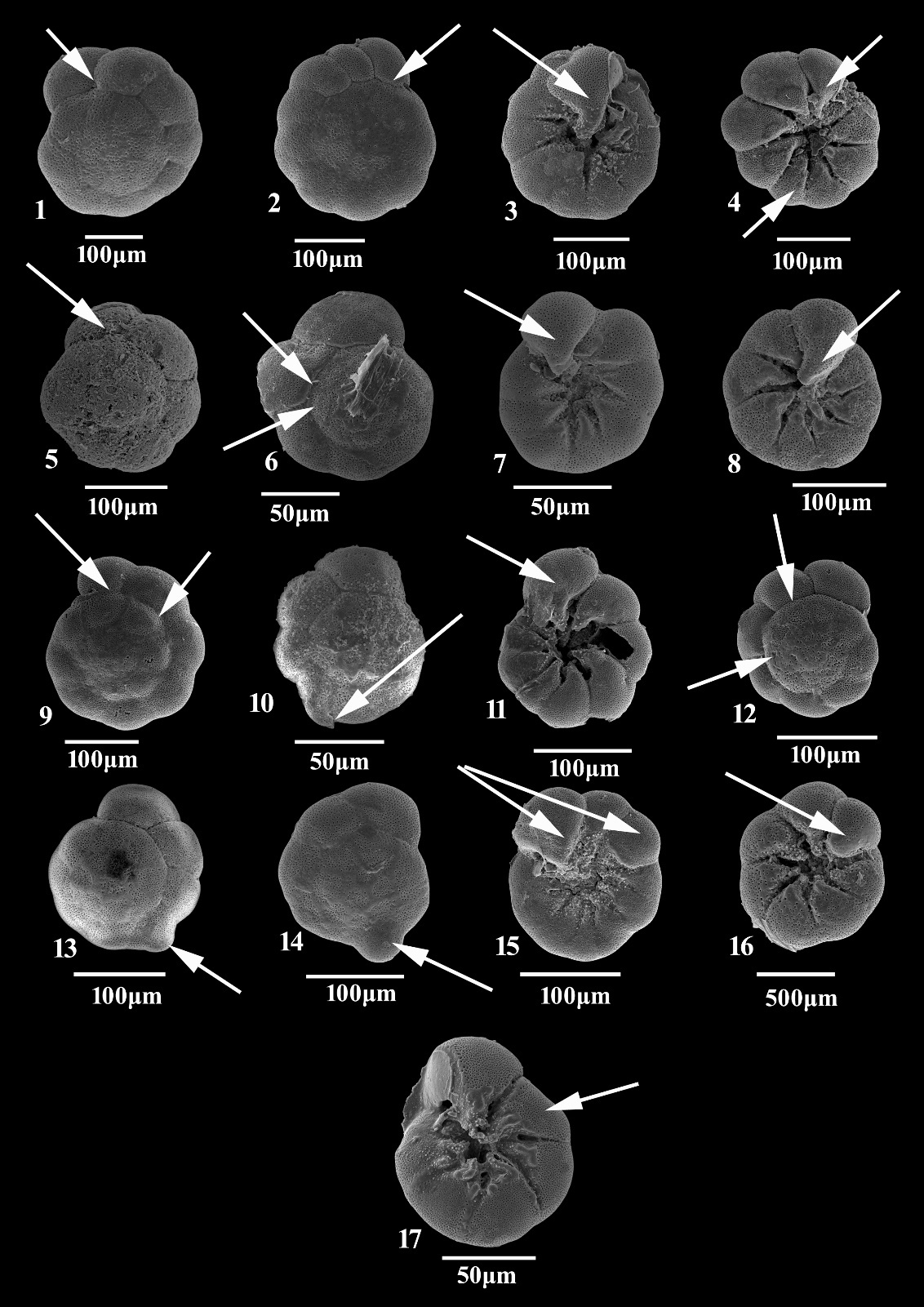
**

**Appendix 3.** Degree of deformation of *Ammonia tepida* (Cushman) Group A: 1. Core IV (0-5cm) 2. Core IV (5-15cm) 3. Core IV (5-15cm) 4-5. Core V (0-5cm) 6-9. Core VI (0-5cm) 10-11. Core V (5-15cm) 12. Core V (15-25cm) 13. Core VII (0-5cm) 14. Core VII (15-25cm) 15. Core VIII (15-25cm) 16. Core VIII (25-35cm) 17. Core IX (25-35cm).

**
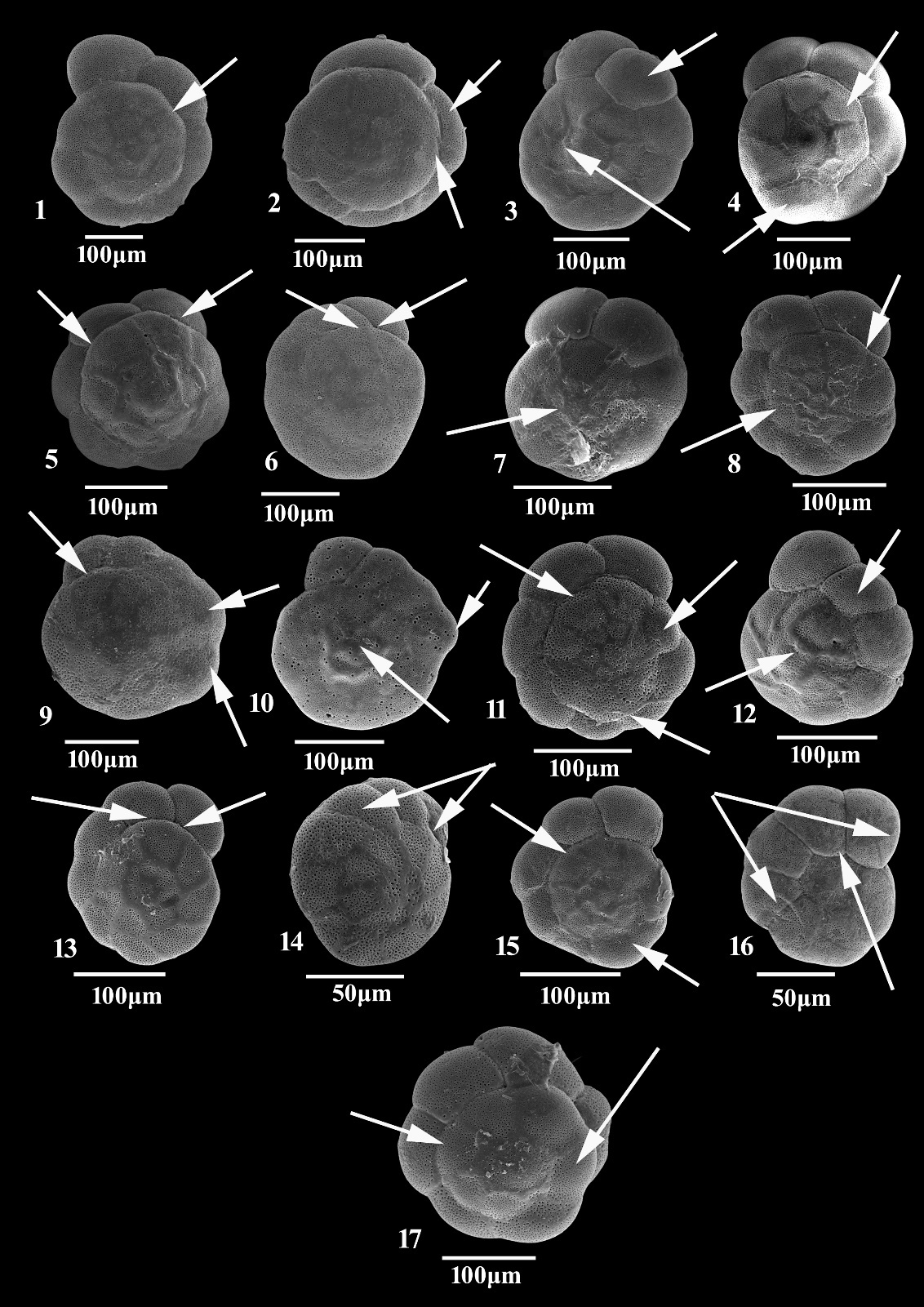
**

**Appendix 4.** Degree of deformation of *Ammonia tepida* (Cushman) Group B: 1. Core IV (0-5cm) 2. Core IV (5-15cm) 3-4. Core V (0-5cm) 5. Core VI (0-5cm) 6. Core VI (5-15cm) 7-9. Core VII (5-15cm) 10-12. Core VII (5-15cm) 13-17. Core VIII (0-5cm).

**
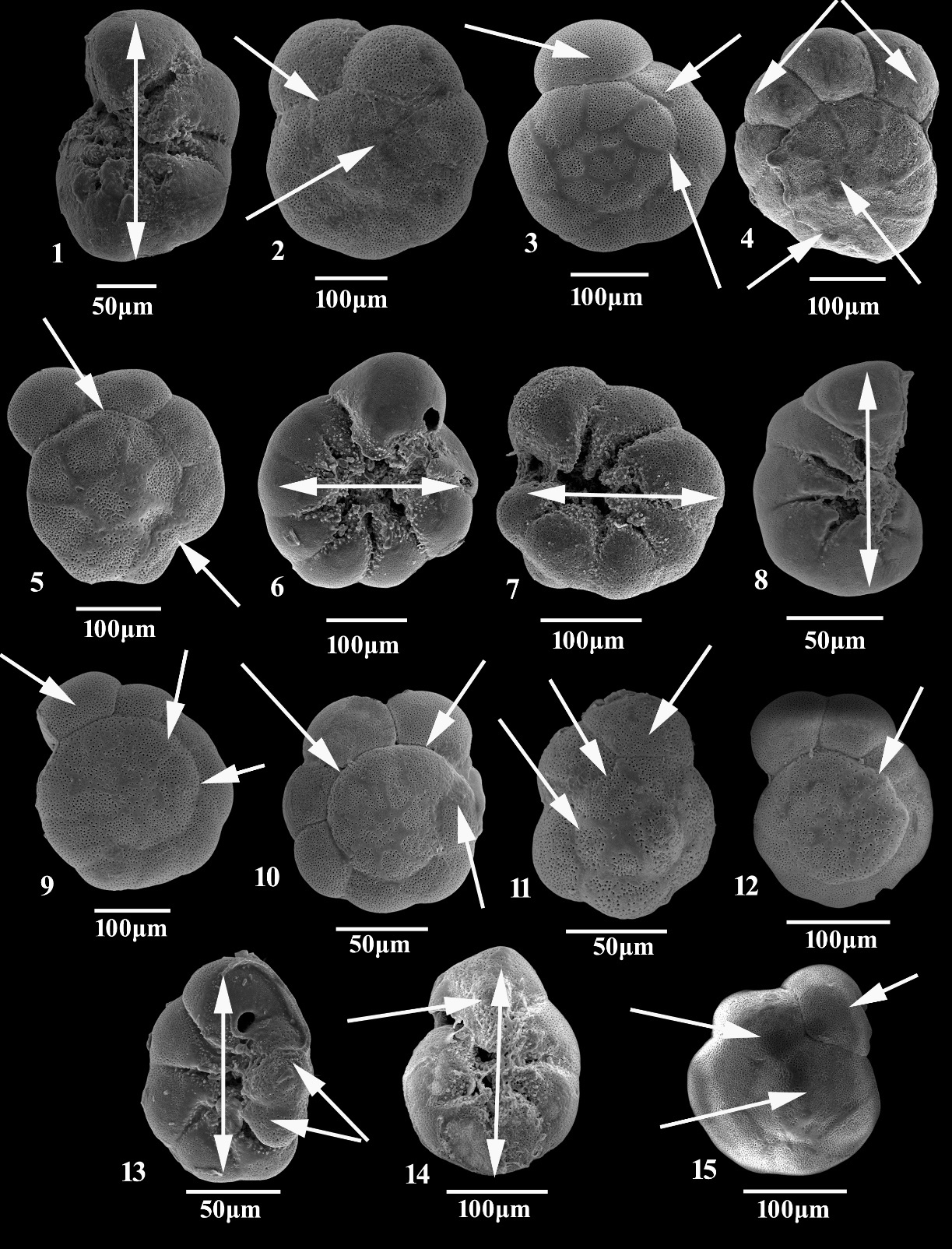
**

**Appendix 5.** Degree of deformation of *Ammonia tepida* (Cushman) Group B: 1-3. Core VIII (5-15cm) 4-5. Core VIII (15-25cm) 6-8. Core IX (0-5cm) 9-11. Core IX (5-15cm) 12-15. Core IX (15-25cm).

**
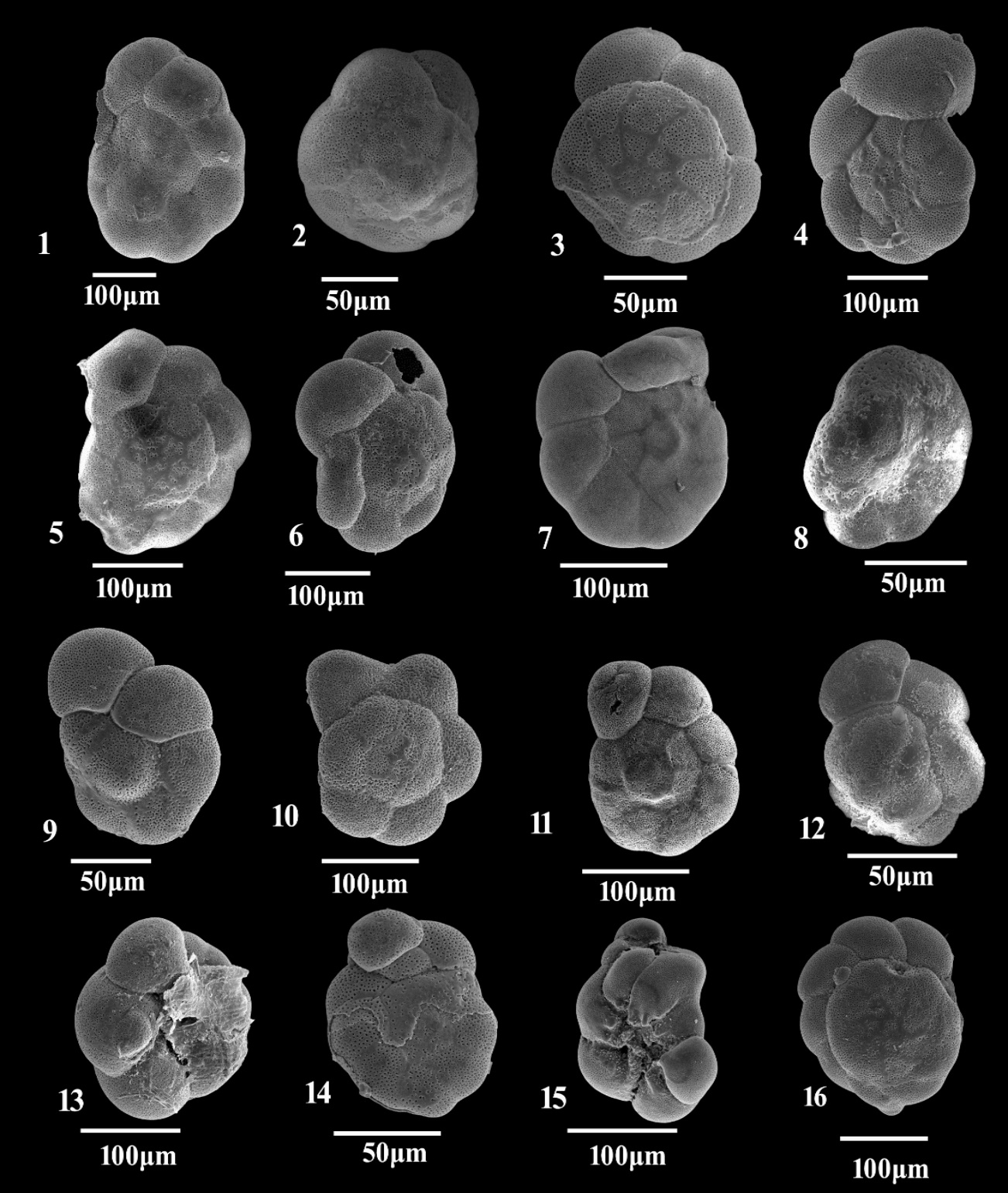
**

**Appendix 6.** Degree of deformation of *Ammonia tepida* (Cushman) Group C: 1-3. Core VII (0-5cm) 4-6. Core VIII (0-5cm) 7. Core VIII (5-15cm) 8-13. Core IX (0-5cm) 14-16. Core IX (5-15cm).

Appendix 7. Varimax rotated component loadings of the three significant components recorded for each core of Edku Lake.

| Variables | PC 1 | PC 2 | PC 3 |
| --- | --- | --- | --- |
| *Q. auberiana* | -0.18 | 0.97 | -0.09 |
| *Q. seminulum* | -0.27 | -0.01 | -0.94 |
| *C. refulgens* | -0.22 | 0.95 | 0.04 |
| *A. mamilla* | -0.29 | 0.90 | -0.21 |
| *A. parkinsonina* | -0.18 | 0.98 | 0.02 |
| *A. tepida* | 0.28 | -0.85 | 0.43 |
| *C. excavatume* | -0.30 | 0.73 | -0.61 |
| S% | -0.76 | 0.40 | -0.42 |
| Z% | 0.53 | -0.47 | 0.51 |
| C% | 0.92 | -0.26 | 0.25 |
| TCO_3_% | 0.78 | 0.41 | -0.18 |
| TOC% | 0.73 | -0.46 | 0.32 |
| Cu | 0.85 | -0.27 | 0.41 |
| Zn | 0.92 | -0.25 | 0.07 |
| Pb | 0.75 | -0.35 | 0.36 |
| Cd | 0.72 | -0.61 | 0.28 |
| Cr | 0.80 | -0.40 | 0.36 |
| Ni | 0.82 | -0.34 | 0.17 |
| As | 0.58 | -0.67 | 0.44 |
| Salinity‰ | -0.50 | 0.74 | -0.44 |
| Eigenvalues | 14.11 | 3.14 | 1.25 |
| Variance % | 70.60 | 15.68 | 6.26 |
| Cumulative Variance % | 70.60 | 86.28 | 92.53 |
